# A Survey of Tables of Probability Distributions

**DOI:** 10.6028/jres.110.007

**Published:** 2005-02-01

**Authors:** Raghu Kacker, Ingram Olkin

**Affiliations:** National Institute of Standards and Technology, Gaithersburg, MD 20899-8910; Stanford University, Stanford, CA 94305-4065

**Keywords:** continuous univariate distributions, discrete univariate distributions, multivariate distributions, probability distributions

## Abstract

This article is a survey of the tables of probability distributions published about or after the publication in 1964 of the Handbook of Mathematical Functions, edited by Abramowitz and Stegun

## 1. Introduction

Probabilities and percentiles of statistical probability distributions have historically been cited from reference tables published in books, journals, and other publications. Reference tables of probability distributions continued to be published from the 1920s through the 1980s and early 1990s. Some tables superceded their earlier counterparts. Abramowitz and Stegun [1] surveyed the tables published before 1964, and reproduced some of them. In particular, Abramowitz and Stegun [1] reproduced the tables of percentiles of chi-square, t-, and F-distributions from the 1954 edition of Pearson and Hartley [2]. Other collections of tables of probability distributions include Greenwood and Hartley [3] and Owen [4].

This article is a survey of the tables published about or after 1964. A few earlier tables are also mentioned when appropriate. Most of the tables abstracted in this article are referenced in Pearson and Hartley [2], Pearson and Hartley [5], Johnson, Kotz, and Kemp [6], Johnson, Kotz, and Balakrishnan [7], Johnson, Kotz, and Balakrishnan [8], Johnson, Kotz, and Balakrishnan [9], and Kotz, Balakrishnan, and Johnson [10]. The abstracts presented here have been verified from the original sources, and in some cases corrections and additions were made. The next three sections contain the abstracts for discrete univariate, continuous univariate, and multivariate probability distributions.

A random variable is denoted by *X*, and *x* denotes a particular value of *X*. The cumulative distribution function of *X* is *F*(*x*) = Pr{*X* ≤ *x*}. The survival function of *X* is $\bar{F}(x)$ = 1−*F*(*x*) = Pr{X > *x*}. For a discrete random variable *f*(*x*) interpreted as Pr{*X* = *x*} is the probability mass function (pmf). For a continuous random variable *f*(*x*) interpreted as d*F*(*x*)/d*x* is the probability density function (pdf). A particular value, *x*, is the rth quantile of *X* when *F*(*x*) = *r*, for 0 ≤ *r* ≤ 1. The rth quantile is commonly referred to as the *r* × 100th percentile of *X*. The expected value or mean, and the variance of *X* are denoted by *E*(*X*), and *V*(*X*), respectively. The abbreviation *n*D, for an integer *n*, denotes *n* decimal places. An expression such as 0.01(0.02)0.09 denotes the sequence of numbers from 0.01 to 0.09 increasing in steps of 0.02. Log denotes natural logs unless indicated otherwise.

## 2. Discrete Univariate Distributions

### 2.1 Binomial Distribution

The pmf is

$$f(x)=\left(\begin{array}{c} n\\ x \end{array}\right)p^{x}{\left(1-p\right)}^{n-x}$$

for *x* = 0, 1, …, *n*.

Weintraub [11] tabulated, to 10D, $\bar{F}(x-1)$ for *p* = 0.0001(0.0001) 0.0009, 0.001(0.001)0.1, and *n* = 1(1)100.

Pearson and Hartley [2] tabulated, to 5D, *f*(*x*) for *p* = 0.01, 0.02(0.02)0.10, 0.10(0.10)0.50, and *n* = 5(5)30.

### 2.2 Poisson Distribution

The pmf is

$$f(x)=\frac{e^{-\theta }\theta^{x}}{x!}$$

for *x* = 0, 1, ….

Defense Systems Department, General Electric Company [12] tabulated, to 8D, *f*(*x*), *F*(*x*), and $\bar{F}(x-1)$ for *θ* ranging from 10^−7^ to 205 with increments ranging from 10^−7^ to 5.

Khamis and Rudert [13] tabulated, to 10D, $\bar{F}(x-1)$ for *θ* = 0.00005(0.00005)0.0005, 0.0005(0.0005)0.005, 0.005(0.005)0.5, 0.5(0.025)3, 3(0.05)8, 8(0.25)33, 33(0.5)83, and 83(1)125.

Pearson and Hartley [2] tabulated, to 6D, *f*(*x*) for *θ* = 0.1(0.1)15.0. They also tabulated, to 5D, *F*(*x*) for *θ* = 0.0005(0.0005)0.005, 0.005(0.005)0.05, 0.05(0.05)1, 1(0.1)5, 5(0.25)10, 10(0.5)20, 20(1)60, and *x* = 1(1)35.

### 2.3 Negative Binomial Distribution

The pmf is

$$f(x)=\left(\begin{array}{c} k+x-1\\ k-1 \end{array}\right)p^{k}{\left(1-p\right)}^{x}$$

for *x* = 0, 1, … and *E*(*X*) = *k*(1 − *p*)/*p*.

Grimm [14] tabulated, to 5D, *f*(*x*) and *F*(*x*) for *E*(*X*) = 0.1(0.1)1.0, 1.0(0.2)4.0, 4.0(0.5)10.0, and 1/*p* = 1.2, 1.5, 2.0(1)5.

Williamson and Bretherton [15] tabulated, to 6D, *f*(*x*) and *F*(*x*) for the following values of *p* and *k*: *p* = 0.05 and *k* = 0.1(0.1)0.5, *p* = 0.10 and *k* = 0.1 (0.1)1.0, *p* = 0.12(0.02)0.20 and *k* = 0.1(0.1)2.5, *p* = 0.22(0.02)0.40 and *k* = 0.1(0.1)2.5(0.5)5.0, *p* = 0.42 (0.02)0.60 and *k* = 0.1(0.1)2.5(0.5)10.0, *p* = 0.62 (0.02)0.80 and *k* = 0.2(0.2)5.0(1)20, *p* = 0.82 (0.02)0.90 and *k* = 0.5(0.5)10.0(2)50, *p* = 0.95 and *k* = 2(2)50(10)200. Deahl [16] extended the Williamson and Bretherton [15] table of *F*(*x*) for *p* = 0.02, 0.04, 0.05, 0.06, 0.08, 0.10, and *k* = 0.10 (0.10)2.00.

Brown [17] tabulated, to 4D, *f*(*x*) and *F*(*x*) for *E*(*X*) = 0.25(0.25)1.0, 1.0(1)10, and 1/*p* = 1.5 (0.5)5.0, 5(1)7.

### 2.4 Hypergeometric Distribution

The pmf is

$$f(x)=\left(\begin{array}{c} k\\ x \end{array}\right)\left(\begin{array}{c} N-k\\ n-x \end{array}\right)/\left(\begin{array}{c} N\\ n \end{array}\right)$$

for max[0, *n* − *N* + *k*] ≤ *x* ≤ min[*k*, *n*].

Lieberman and Owen [18] tabulated, to 6D, *f*(*x*) and *F*(*x*) for *N* = 2(1)100, *n* = 1(1)50, and all possible values of *k*; *N* = 1000, *n* = 500, and all possible values of *k*; and *N* = 100(100)2000, *n* = *N*/2, and *k* = (*n* − 1).

### 2.5 Logarithmic Series Distribution

The pmf is

$$f(x)=\frac{-\theta^{x}}{\left[\log(1-\theta )\right]x}$$

for *x* = 1, 2, … and *E*(*X*) = − *θ*/[(1 − *θ*) log(1 − *θ*)].

Patil [19] tabulated, to 4D, *E*(*X*) as a function of *θ* for *θ* = 0.01(0.01)0.99. Patil, Kamat, and Wani [20] tabulated, to 6D, *f*(*x*) and *F*(*x*) for *θ* = 0.01(0.01)0.70, 0.70(0.005)0.900, and 0.900(0.001)0.999. Patil and Wani [21] tabulated, to 4D, parameter *θ* for *E*(*X*) = 1.02(0.02)2.00, 2.00(0.05)4.00, and 4.00(0.1)8.0, 8.0(0.2)16.0, 16.0(0.5)30.0, 30.0(2)40, 40(5)60, 60(10)140, and 140(20)200.

Williamson and Bretherton [22] tabulated, to 5D, *f*(*x*) and *F*(*x*) for *E*(*X*) = 1.1(0.1)2.0, 2.0(0.5)5.0, 5.0(1)10.0. They also tabulated *θ*, to 5D, for *E*(*X*) = 1.0(0.1)10.0, and 10.0(1)50.

### 2.6 Neyman Type A Distribution

This is the Poisson-stopped-summed-Poisson distribution (Johnson, Kotz, and Kemp [6]). The pmf is

$$f(x)=\frac{e^{-\lambda }\phi^{x}}{x!}\sum_{j=0}^{\infty }\frac{{\left(\lambda e^{-\phi }\right)}^{j}j^{x}}{j!}$$

for *x* = 0, 1, … and *E*(*X*) = *λϕ*.

Grimm [23] tabulated, to 5D, *f*(*x*) for *E*(*X*) = 0.1(0.1)1.0, 1.0(0.2)4, 6, 10, and ø = 0.2, 0.5, 1.0, 2, 3, 4 up to *f*(*x*) = 0.99900.

### 2.7 Geometric-Poisson Distribution

The pmf is

$$f(x)=e^{-\theta }p^{x}\sum_{j=1}^{x}\left(\begin{array}{c} x-1\\ j-1 \end{array}\right)\frac{{\left(\theta (1-p)/p\right)}^{j}}{j!}$$

for *x* = 1, 2, … ; *f*(*x*) = e^−^*^θ^* for *x* = 0; and *E*(X) = *θ*/(1−*p*). This distribution is also called Pólya-Aeppli distribution.

Sherbrooke [24] tabulated, to 4D, *f*(*x*) and *F*(*x*) for *E*(*X*) = 0.10, 0.25(0.25)1.00, 1.00(0.5)3.0, 3.0(1)10, and (1 + *p*)/(1 − *p*) = 1.5(0.5)5.0, 5.0(1)7.

## 3. Continuous Univariate Distributions

### 3.1 Standard Normal (Gaussian) Distribution

The pdf is

$$f(x)=\frac{1}{\sqrt{2\pi }}e^{-x^{2}/2}.$$

Abramowitz and Stegun [1] tabulated the following: *F*(*x*), to 15D, for *x* = 0.00(0.02)3.00 and, to 10D, for *x* = 3.00(0.05)5.00; *f*(*x*), to 5D, for *x* such that $\bar{F}(x)$ = *q* for *q* = 0.000(0.001)0.500; and *x* such that $\bar{F}(x)$ = *q* for *q* = 0.000(0.001)0.500, 0.0000(0.0001) 0.0250, and *q* = 10^−^*^m^* for *m* = 4(1)23. They also tabulated the derivatives of *f*(*x*) up to the order 12.

Pearson and Hartley [5] tabulated, to 10D, quantiles *x* and corresponding *f*(*x*), where *F*(*x*) = *p* for *p* = 0.500(0.001)0.999, and 0.9990(0.0001)0.9999.

White [25] tabulated, to 20D, quantiles *x* such that $\bar{F}(x)$ = *q* for *q* = 0.005(0.005)0.500, and *q* = 5 × 10^−^*^k^*, 2.5 × 10^−^*^k^*, and 1 × 10^−^*^k^*, where *k* = 1(1)20.

### 3.2 Standardized Stable Distributions

The pdfs of standardized stable distributions are unimodal with shape depending on the parameters *β* and *α*. Although the pdfs are rather complicated, they can be expressed as convergent series (Johnson, Kotz, and Balakrishnan [7]).

Fama and Roll [26] tabulated, to 4D, *F*(*x*) for *β* = 0 and *α* = 1.0(0.1)1.9, 1.95, 2.0, and *x* = 0.05(0.05)1.00, 1.00(0.1)2.0, 2.0(0.2)4.0, 4.0(0.4)6.0, 6.0(1)8, 10, 15, and 20. They also tabulated, to 3D, quantiles *x* such that *F*(*x*) = *p*, for *p* = 0.52(0.02)0.94, 0.94(0.01)0.97, 0.97(0.005)0.995, and 0.9975.

Holt and Crow [27] tabulated, to 4D, *f*(*x*) for *β* = − 1.00(0.25)1.00 and α = 0.25(0.25)2.00, and nonnegative *x* in steps varying by factors of 10 from 0.001 to 100 such that interpolation is possible. The tabulation is terminated when *f*(*x*) first falls to 0.0001. The largest such value of *x* is 338, for *α* = 0.25 and *β* = − 1.00.

Worsdale [28] tabulated, to 4D, *F*(*x*) for *β* = 0 and *α* = 0.6(0.1)2.0, and *x* = 0.00(0.05)3.00. For larger values of *x*, *F*(*x*) is tabulated for log_10_ *x* = 0.40(0.05)2.50.

Panton [29] tabulated, to 5D, *F*(*x*) for *β* = 0 and *α* = 1.0(0.1)2.0, and *x* = 0.05(0.05)1.00, 1.00(0.1)2.0, 2.0(0.2)4.00, 4.00(0.4)6.0, 7, 8, 10, 15, 20.

### 3.3 Inverse Gaussian Distribution

The pdf is

$$f(x)={\left(\frac{\lambda }{2\pi x^{3}}\right)}^{1/2}\;\exp\left(\frac{-\lambda {\left(x-\mu \right)}^{2}}{2\mu^{2}x}\right)$$

for *x* > 0, *λ* > 0, *µ* > 0, and *E*(*X*) = *µ* and *V*(*X*) = *µ*^3^/*λ*.

Wasan and Roy [30] tabulated, to 4D, quantiles *x* such that *µ* = *t*, *λ* = *t*^2^, that is, *µ* = *t* = *V*(*X*), and *F*(*x*) = *p*, where *t* = 0.1(0.1)4.0, 4.0(0.2)6.0, 6.0(1.0)35.0, 35(5)100, 100(10)150, 150(20)250, 300(100)1000, 1000(200)1600, 2000(400)4000, and *p* = 0.005, 0.010, 0.025(0.025)0.100, 0.25(0.25)0.75, 0.80, 0.900(0.025)0.975, and 0.990. In order to determine quantiles of an inverse Gaussian random variable *Y* with parameters *µ* > 0 and *λ* > 0, use the fact that the distribution of *X* = *λ Y*/*µ*^2^ is inverse Gaussian with parameters *t* and *t*^2^, where *t* = *λ*/*µ*.

Chan, Cohen, and Whitten [31] tabulated *F*(*x*) of the standardized inverse Gaussian distribution with *E*(*X*) = 0 and *V*(*X*) = 1 for various values of the standardized third moment about the mean $\alpha_{3}=\sqrt{\beta_{1}}$. They tabulated, to 6D, *F*(*x*) for *x* = −3.0(0.1)5.9 with *α*_3_ = 0.0(0.1)1.2, and *x* = −1.5(0.1)7.4 with *α*_3_ = 1.3(0.1)2.5.

Koziol [32] tabulated quantiles *x*, to eight significant digits, such that *F*(*x*) = *p*, *µ* = *t*, *λ* = *t*^2^, where *t* = 0.02(0.02)4, 4(0.04)6, 6(0.02)35, 35(1)100, 100(2)150, 150(4)250, 250(10)300, 300(20)600, 600(40)2000, 2000(80)4000, and *p* = 0.001, 0.005, 0.01(0.01)0.99, 0.995, 0.999.

### 3.4 Incomplete Gamma Function

Harter [33] tabulated, to 9D, the incomplete Γ-function ratio *I*(*u*, *p*) defined by Pearson [34] as

$$I(u,\;p)=\frac{1}{\Gamma (p+1)}\int_{0}^{u\sqrt{p+1}}v^{p}e^{-v}dv$$

for *u* at intervals of 0.1, starting from 0.0, and *p* = − 0.5(0.5)74 and 74(1)164. Harter [35] extended Harter [33] for *p* = − 0.95(0.05)4.

### 3.5 Standard Gamma Distribution

The pdf is

$$f(x)=\frac{1}{\Gamma (\alpha )}x^{\alpha -1}e^{-x}$$

for *x* ≥ 0 and *α* > 0.

Wilk, Gnanadesikan, and Huyett [36] tabulated quantiles *x*, accurate to about five significant digits, for *α* = 0.1(0.1)0.6, 0.6(0.2)5.0, 5.0(0.5)10.0, 10.0(1.0)20.0, and *p* = 0.1, 0.5, 0.7, 1.0(0.5)3.0, 3.0(1.0)5.0, 7.5, 10.0(5.0)30.0, 30(10)70, 70(5)90, 90.0(2.5)97.5, 98.0, 99.0, 99.5, and 99.9.

Thom [37] tabulated, to 4D, *F*(*x*) for *α* = 0.5(0.5)15.0, 15(1)36, and *x* = 0.0001, 0.001, 0.004(0.002)0.020, 0.02(0.02)0.80, 0.8(0.1)2.0, 2.0(0.2)3.0, 3.0(0.5)9.0; also tabulated, to 4D, quantiles *x* and corresponding *f*(*x*) such that *F*(*x*) = *p* for *α* = 0.5(0.5)15.0, 15(1)36, and *p* = 0.01, 0.05(0.05)0.95, 0.99.

Harter [38] tabulated, to 5D, quantiles *x* such that *F*(*x*) = *p* against the coefficient of skewness $\sqrt{\beta_{1}}=\mu_{3}/\sigma^{3}=\;2/\sqrt{\alpha }$ for = $\sqrt{\beta_{1}}$= 0.0(0.1)4.8, and 4.8(0.2)9.0, and *p* = 0.0001, 0.0005, 0.0010, 0.0050, 0.0100, 0.0200, 0.0250, 0.0400, 0.0500, 0.1000(0.1000)0.9000, 0.9500, 0.9600, 0.9750, 0.9800, 0.9900, 0.9950, 0.9990, 0.9995 and 0.9999. Harter [39] extended Harter [38] for *p* = 0.002000, 0.429624, 0.570376, and 0.998000.

### 3.6 Chi-Square Distribution

The pdf is

$$f(x)=x^{(v-2)/2}/\left[2^{v/2}\Gamma (v/2)e^{-x/2}\right]$$

for *x* > 0 and degrees of freedom *ν* > 0. If *X*_1_, *X*_2_, …, *X_ν_* have independent standard normal distributions, then $X=\sum_{i=1}^{n}X_{i}^{2}$ has a chi-square distribution with *ν* degrees of freedom.

Harter [33] tabulated, to six significant digits, quantiles *x* such that *F*(*x*) = *p* for *p* = 0.0001, 0.0005, 0.001, 0.005, 0.01, 0.025, 0.05, 0.1(0.1)0.9, 0.95, 0.975, 0.99, 0.995, 0.999, 0.9995, and 0.9999, and *ν* = 1(1)150, and 150(2)330. A subset of these tables for *ν* = 1(1)100 is reproduced in Harter [40].

Khamis and Rudert [13] tabulated *F*(*x*), to 10D, for *ν* = 0.1(0.1)20, 20(0.2)40, 40(0.5)140, and *x* = 0.0001(0.0001)0.001, 0.001(0.001)0.01, 0.01(0.01)1, 1(0.05)6, 6(0.1)16, 16(0.5)66, 66(1)166, 166(2)250.

Pearson and Hartley [5] tabulated, to six significant digits, quantiles *x* such that *F*(*x*) = *p* for *p* = 0.0001, 0.0005, 0.001, 0.005, 0.01, 0.025, 0.05, 0.1(0.1)0.9, 0.95, 0.975, 0.99, 0.995, 0.999, 0.9995, and 0.9999, and *ν* = 0.1(0.1)3.0, 3.0(0.2)10.0, and 10(1)100.

Mardia and Zemroch [41] tabulated, to five significant digits, quantiles *x* such that *F*(*x*) = *p* for *p* = 0.0001, 0.0005, 0.001, 0.005, 0.01, 0.02, 0.025, 0.03(0.01)0.1, 0.2, 0.25, 0.3(0.1)0.7, 0.75. 0.8, 0.9(0.01).97, 0.975, 0.98, 0.99, 0.995, 0.999, 0.9995, 0.9999, and fractional degrees of freedom *ν* = 0.1(0.1)3.0, 3.0(0.2)7.0, 7.0(0.5)11, 11(1)30, 30(5)60, 60(10)120.

### 3.7 Standardized Weibull Distribution

The pdf is

$$f(x)=\gamma x^{\gamma -1}e^{-x^{\gamma }}$$

for *x* > 0 and *γ* > 0, where *γ* is the shape parameter.

Plait [42] tabulated, to 8D, *f*(*x*) for *γ* = 0.1(0.1)3, 3(1)10, and tabulated, to 7D, *F*(*x*) for *γ* = 0.1(0.1)4.0.

### 3.8 Standardized Extreme Value Distribution —Type 1

The pdf is

$$f(x)=\exp\;(-x-e^{-x}).$$

Gumbel [43] tabulated, to 7D, *f*(*x*) and *F*(*x*) for the following values of *x*: −3.0(0.1) −2.4, −2.40(0.05)0.00, 0.0(0.1)4.0, 4.0(0.2)8.0, and 8.0(0.5)17.0. Also, tabulated, to 5D, quantiles *x* such that *F*(*x*) = *p* for *p* = 0.0001(0.0001)0.0050, 0.005(0.001)0.988, 0.9880(0.0001)0.9994, and 0.99940(0.00001)0.99999.

White [44] tabulated, to 7D, the means and variances of all order statistics for sample sizes 1(1)50 and 50(5)100. Extended tables of means, variances, and covariances of all order statistics for sample sizes up to 30 have been provided by Balakrishnan and Chan [45] and Balakrishnan and Chan [46].

### 3.9 Incomplete Beta Function

Pearson [47] tabulated, to 7D, the incomplete *B*-function ratio *I*(*p*, *q*) defined as

$$I\left(p,q\right)=\frac{\Gamma \left(p+q\right)}{\Gamma \left(p\right)\Gamma \left(q\right)}\int_{0}^{x}t^{p-1}{\left(1-t\right)}^{q-1}dt$$

for *p*, *q* = 0.5(0.5)11.0(1)50 with *p* ≥ *q* and *x* = 0.00(0.01)1.00. These values are reproduced in Pearson [48]. Additional values of *I*(*p*, *q*) are given, to 7D, for *p* = 11.5(1.0)14.5, *q* = 0.5, and *x* = 0.00(0.01)1.00. More values of *I*(*p*, *q*) are given, to 7D, for *p* = 0.5(0.5)11.0(1)16, *q* = 1.0(0.5)3.0, and *x* = 0.975, 0.980, 0.985, 0.988(0.001)0.999. Even more values of *I*(*p*, *q*) are given for *p* = 0.5(0.5)11.0(1)16, *q* = 0.5, and *x* = 0.9750, 0.9800, 0.9850, 0.9880(0.0005)0.9985, 0.9988(0.0001)0.9999. For *x* ≥ 0.988, values are given to 8D.

### 3.10 Beta Distribution

The pdf is

$$f(x)=\frac{1}{B(a,b)}x^{a-1}{\left(1-x\right)}^{b-1}$$

for 0 < *x* < 1, *a* > 0, *b* > 0 and *B*(*a*, *b*) = Γ(*a*)Γ(*b*)/Γ(*a* + *b*).

Harter [33] tabulated, to 7D, quantiles *x* such that *F*(*x*) = *p* for *a* = 1(1)40, *b* = 1(1)40, and *p* = 0.0001, 0.0005, 0.001, 0.005, 0.01, 0.025, 0.05, 0.1(0.1)0.9, 0.95, 0.975, 0.99, 0.995, 0.999, 0.9995, 0.9999.

Vogler [49] tabulated, to six significant digits, quantiles *x* such that *F*(*x*) = *p* for *a* = 0.50(0.05)1.00, 1.1, 1.25(0.25)2.50, 2.50(0.5)5.0, 6, 7.5, 10, 12, 15, 20, 30, 60, *b* = 0.5(0.5)5.0, 6, 7.5, 10, 12, 15, 20, 30, 60, and *p* = 0.0001, 0.001, 0.005, 0.01, 0.025, 0.05, 0.1, 0.25, 0.5.

Pearson and Hartley [2] tabulated, to five significant digits, quantiles *x* such that *F*(*x*) = *p* for *a* = 0.5(0.5)15.0, 20, 30, 60, *b* = 0.5(0.5)5.0, 6, 7.5, 10, 12, 15, 20, 30, 60, and *p* = 0.001, 0.0025, 0.005, 0.01, 0.025, 0.05, 0.10, 0.25, 0.50.

### 3.11 F-Distribution

If *X*_1_ and *X*_2_ have independent chi-square distributions with degrees of freedom *ν*_1_ and *ν*_2_, respectively, then

$$X=\frac{X_{1}/v_{1}}{X_{2}/v_{2}}$$

has an F-distribution with *ν*_1_ (numerator) and *ν*_2_ (denominator) degrees of freedom.

Pearson and Hartley [5] tabulated, to five significant digits, quantiles *x* such that *F*(*x*) = *p* for *p* = 0.5, 0.75, 0.90, 0.95, 0.975, 0.99, 0.995, 0.9975, 0.999, and *ν*_1_ = 1(1)10, 12, 15, 20, 24, 30, 40, 60, 120,∞, and *ν*_2_ = 1(1)30, 40, 60, 120, ∞.

Mardia and Zemroch [41] tabulated, to five significant digits, quantiles *x* such that *F*(*x*) = *p* for *p* = 0.5, 0.6, 0.7, 0.75, 0.8, 0.90(0.01)0.99, 0.975, 0.995, 0.999, 0.9995, 0.9999, and *ν*_1_ = 0.1(0.1)1.0, 1.0(0.2)2.0, 2.0(0.5)5, 5(1)16, 18, 20, 24, 30, 40, 60, 120, ∞, and *ν*_2_ = 0.1(0.1)3.0, 3.0(0.2)7.0, 7.0(0.5)11, 11(1)40, 60, 120, ∞. A part of this table is reproduced in Pearson and Hartley [5].

### 3.12 t-Distribution

If *X*_1_ has the standard normal distribution and *X*_2_ has an independent chi-square distribution with *ν* degrees of freedom, then

$$X=X_{1}/\sqrt{X_{2}/v}$$

has a Student’s t-distributon with *ν* degrees of freedom.

Fisher and Yates [50] tabulated, to 3D, quantiles *x* such that *F*(*x*) = *p* for *p* = 0.55(0.05)0.95, 0.975, 0.99, 0.995, 0.9995, and *ν* = 1(1)30, 40, 60, 120. Lempers and Louter [51] extend these tables for *p* = 0.5625(0.0625)0.9375.

Hill [52] tabulated, to 20D or 20 significant digits, quantiles *x* such that *F*(*x*) = *p*/2 where *p* = 0.9(−0.1)0.1, 10^−^*^m^*, 2 × 10^−^*^m^*, 5 × 10^−^*^m^*, for *m* = 2(1)10(5)30, and *ν* = 1(1)30, 30(2)50, 50(5)100, 100(10)150, 200, [240, 300, 400, 600, 1200] × [1, 10, 100], and ∞.

Mardia and Zemroch [41] tabulated, to five significant digits, quantiles *x* such that *F*(*x*) = *p* for *p* = 0.5, 0.6, 0.7, 0.75, 0.8, 0.90(0.01)0.99, 0.975, 0.995, 0.999, 0.9995, 0.9999, and fractional degrees of freedom *ν* = 0.1(0.1)3.0, 3.0(0.2)7.0, 7.0(0.5)11, 11(1)40, 60, 120, and ∞.

### 3.13 Noncentral Chi-Square Distribution

If *X*_1_, *X*_2_, ⋯, *X_ν_* have independent standard normal distributions and *δ*_1_, *δ*_2_, ⋯, *δ_ν_* are constants then

$$X=\sum_{i=1}^{v}{\left(X_{l}+\delta_{i}\right)}^{2}$$

has a noncentral chi-square distribution with *ν* degrees of freedom and noncentrality parameter $\lambda =\sum_{i=1}^{v}\delta_{i}^{2}$.

Johnson [53] tabulated, to four significant digits, quantiles *x* such that *F*(*x*) = *p* for *p* = 0.001, 0.0025, 0.005, 0.01, 0.025, 0.05, 0.10, 0.25, 0.5, 0.75, 0.90, 0.95, 0.975, 0.99, 0.995, 0.9975, 0.999, *ν* = 1(1)12, 15, 20, and square root of the noncentrality parameter $\sqrt{\lambda }$ = 0.2(0.2)6.0.

Haynam, Govindarajulu, and Leone [54] tabulated the power 1 − *β* of chi-square test of significance as a function of the level of significance *α*, degrees of freedom *ν*, and noncentrality parameter *λ* for *α* = 0.001, 0.005, 0.01, 0.025, 0.05, 0.1, *ν* = 1(1)30, 30(2)50, 50(5)100, and *λ* = 0.0(0.1)1.0, 1.0(0.2)3.0, 3.0(0.5)5.0, 5(1)40, 40(2)50, 50(5)100. They also tabulated the noncentrality parameter *λ* as a function of *α*, *ν*, and 1 − *β* for the values of *α* and *ν* listed above and 1 − *β* = 0.1(0.02)0.7, 0.7(0.01)0.99.

Pearson and Hartley [5] tabulated, to 3D, noncentrality parameter *λ* as a function of the level of significance *α*, degrees of freedom *ν*, and power 1 − *β* for *α* = 0.05, 0.01, *ν* = 1(1)30, 30(2)50, 50(5)100, and 1 − *β* = 0.25, 0.50, 0.60, 0.70(0.05)0.95, 0.97, 0.99.

### 3.14 Noncentral Chi Distribution

If *X*_1_ has a noncentral chi-square distributon then the distribution of $X=\sqrt{X_{1}}$ is referred to as noncentral chi distribution.

Johnson and Pearson [55] tabulated, to four significant digits, quantiles *x* of chi distribution such that *F*(*x*) = *p* for *p* = 0.005, 0.01, 0.025, 0.05, 0.95, 0.975, 0.99, 0.995, degrees of freedom *ν* = 1(1)12, 15, 20, and square root of the noncentrality parameter $\sqrt{\lambda }$ = 0.0(0.2)6.0. Approximate quantiles to three significant digits are also given for $\sqrt{\lambda }$ = 8.0 and 10.0. These tables are reproduced in Pearson and Hartley [5].

### 3.15 Noncentral F-Distribution

If *X*_1_ has a noncentral chi-square distribution with *ν*_1_ degrees of freedom and noncentrality parameter *λ*, *X*_2_ has a chi-square distribution with *ν*_2_ degrees of freedom, and *X*_1_ and *X*_2_ are independently distributed then

$$X=\frac{X_{1}/v_{1}}{X_{2}/v_{2}}$$

has a noncentral F-distribution with *ν*_1_ and *ν*_2_ degrees of freedom and noncentrality parameter *λ*.

Tiku [56] tabulated, to 4D, the power of the F-test for the level of significance *α* = 0.005, 0.01, 0.025, 0.05, *ν*_1_ = 1(1)10, 12, and *ν*_2_ = 2(2)30, 40, 0, 120, ∞, and noncentrality parameter *λ* such that $\sqrt{\lambda /(v_{1}+1)}$ = 0.5, 1.0(0.2)2.2, 2.2(0.4)3.0.

### 3.16 Doubly Noncentral F-Distribution

If *X*_1_ has a noncentral chi-square distribution with *ν*_1_ degrees of freedom and noncentrality parameter *λ*_1_, *X*_2_ has a noncentral chi-square distribution with *ν*_2_ degrees of freedom and noncentrality parameter *λ*_2_, and *X*_1_ and *X*_2_ are independently distributed then

$$X=\frac{X_{1}/v_{1}}{X_{2}/v_{2}}$$

has a doubly noncentral F-distribution with *ν*_1_ and *ν*_2_ degrees of freedom, and noncentrality parameters *λ*_1_ and *λ*_2_.

Tiku [57] tabulated, to 4D, the power of the F-test for the level of significance *α* = 0.01 and 0.05, degrees of freedom *ν*_1_ = 1(1)8, 10, 12, 24 and *ν*_2_ = 2(2)12, 16, 20, 24, 30, 40, 60, noncentrality parameters *λ*_1_ and *λ*_2_ such that $\phi_{1}=\sqrt{\lambda_{1}/(v_{1}+1)}=0(0.5)3$ and $\phi_{2}=\lambda_{2}/\sqrt{v_{2}}$. Tiku [57] also tabulated, to 4D, the power of F-test for the critical values *F*_0_ such that *u*_0_ = 1/[1 + (*ν*_1_/*ν*_2_)*F*_0_] = 0.02(0.08)0.50, 0.60, 0.75, 0.95, degrees of freedom *ν*_1_ = *ν*_2_ = 4(2)12, and the same noncentrality parameters as used in the previous table.

### 3.17 Noncentral t-Distribution

If *X*_1_ has the standard normal distribution and *X*_2_ has an independent chi-square distribution with *ν* degrees of freedom, then

$$X=(X_{1}+\delta )/\sqrt{X_{2}/v}$$

has a noncentral t-distributon with *ν* degrees of freedom and noncentrality parameter *δ*.

Bagui [58] tabulated, to 5D, quantiles *x* of noncentral t-distribution such that *F*(*x*) = *p* for *p* = 0.01, 0.025, 0.05, 0.10, 0.20, 0.30, 0.70, 0.80, 0.90, 0.95, 0.975, 0.99, degrees of freedom *ν* = 1(1)60, and noncentrality parameter *δ* = 0.1(0.1)8.0.

### 3.18 Doubly Noncentral t-Distribution

If *X*_1_ has the standard normal distribution and *X*_2_ has an independent noncentral chi-square distribution with *ν* degrees of freedom and noncentrality parameter *λ*, then

$$X=(X_{1}+\delta )/\sqrt{X_{2}/v}$$

has a doubly noncentral t-distributon with *ν* degrees of freedom, numerator noncentrality parameter *δ*, and denominator noncentrality parameter *λ*.

Bulgren [59] tabulated, to 6D, *F*(*x*) of doubly noncentral t-distribution with degrees of freedom *ν* = 2(1)20, absolute value of numerator noncentrality parameter |*δ*| = 0(1)5, denominator noncentrality parameter *λ* = 0, 1, 2(2)8, and *x* = 0.0, 0.1, 0.2(0.2)9.0.

### 3.19 Distribution of the Sample Correlation Coefficient From Bivariate Normal Distribution

Suppose (*Y_i_*, *Z_i_*), for *i* = 1, 2, …, *n*, are independently distributed and have a common joint bivariate normal distribution with correlation coefficient *ρ*. Then the sample correlation coefficient

$$X=\frac{\sum_{i=1}^{n}\left(Y_{i}-\bar{Y})(Z_{i}-\bar{Z}\right)}{\sqrt{{\sum_{i=1}^{n}{\left(Y_{i}-\bar{Y}\right)}^{2}\sum_{i=1}^{n}\left(Z_{i}-\bar{Z}\right)}^{2}}}$$

has a distribution that depends only on the correlation coefficient *ρ* and the sample size *n*.

Odeh [60] tabulated, to 5D, quantiles *x* of the sample correlation coefficient, where *F*(*x*) = *p* for *p* = 0.005, 0.01, 0.025, 0.05, 0.10, 0.25, 0.75, 0.90, 0.95, 0.975, 0.99, 0.995, *ρ* = 0.0(0.10)0.90, 0.95, and *n* = 4(1)30, 30(2)40, 40(5)50, 50(10)100, 100(20)200, and 200(100)1000.

### 3.20 Distribution of the Sample Multiple Correlation Coefficient From Multivariate Normal Distribution

If the random variables *X*_1_, …, *X_M_* have a joint multivariate normal distribution, then the smallest mean squared error linear predictor of *X*_1_ is the conditional expected value *E*(*X*_1_|*x*_2_, …, *x_M_*). The multiple correlation coefficient *R* is the correlation between *X*_1_ and its smallest mean squared error linear predictor. The distribution of the sample multiple correlation coefficient *r* depends only on the population coefficient *R*, number of variates *M*, and the sample size *N*.

Pearson and Hartley [5] tabulated, to 3D, lower and upper 1 and 5 percent points of the sample multiple correlation coefficient for *R* = 0.1(0.1)0.9, the sample size *N* such that *N* − *M* = 10(10)50, and *M* − 1 = 2(2)12, 12(4)24, 30, 34, 40.

## 4. Multivariate Distributions

### 4.1 Multivariate Normal Distribution

The multivariate normal density function of the random vector (*X*_1_, …, *X_M_*) is

$$f\left(x_{1},\ldots ,x_{M}\right)=\frac{|\Sigma {|}^{-1/2}}{{\left(2\pi \right)}^{M/2}}\;\exp\left[-(1/2)(x-\mu {)}^{\prime }\sum^{-1}(x-\mu )\right],$$

where ***x*** = (*x*_1_, …, *x_M_*)′, ***µ*** = (*µ*_1_, …, *µ_M_*)′ is the mean vector, and ***Σ*** = [*σ_ij_*] is the positive definite covariance matrix. Here (*x*_1_, …, *x_M_*)′, denotes transpose of the vector (*x*_1_, …, *x_M_*). For the case ***µ*** = (0, …, 0)′, *σ_ii_* = 1, *σ_ij_* = *ρ*, where 0 ≤ *ρ* < 1 and *i*, *j* = 1, …, *M*, *i* ≠ *j*, a one-sided upper equicoordinate *p* × 100 percentage point *g* is such that

$$\mathrm{Pr}\left\{\underset{1\leq i\leq M}{\max}X_{i}\leq g\right\}=p,$$

and a two-sided upper equicoordinate *p* × 100 percentage point *h* is such that

$$\mathrm{Pr}\left\{\underset{1\leq i\leq M}{\max}\left|X_{i}\right|\leq h\right\}=p,$$

where *p* is a specified value for the probability integral.

Gupta [61] tabulated equicoordinate one-sided probabilities *p*, to 5D, for *g* = −3.5(0.1)3.5, *M* = 1(1)12, and *ρ* = 0.100, 0.125, 0.200(0.05)0.300, 1/3, 0.375, 0.400(0.1)0.600, 0.625, 2/3, 0.700(0.05)0.800, 0.875, and 0.900.

Tong [62] tabulated equicoordinate one-sided and two-sided percentage points, to 4D, and probability integrals *p*, to 5D. The table of one-sided percentage points gives the values of *g* for *M* = 2(1)20, *ρ* = 0.0(0.1)0.9, 1/3, 2/3, 1/4, and 3/4, and *p* = 0.90, 0.95, and 0.99. The table of one-sided probability integrals gives the values of *p* for *g* = −2.0(0.1)4.0, *M* = 2(1)10, 10(2)20, and *ρ* = 0.0(0.1)0.9, 1/3, 2/3, 1/4, and 3/4. The table of two-sided percentage points gives the values of *h* for the same set of *M*, *ρ*, and *p* as the one-sided percentage points. The table of two-sided probability integrals gives the values of *p* for *h* = 0.1(0.1)5.0 and the same set of *M*, and *ρ* as the one-sided probability integrals.

### 4.2 Multivariate t-Distribution

Suppose the random vector ***Z*** = (*Z*_1_, …, *Z_M_*)′ has a multivariate normal distribution with mean ***µ*** = **0** and covariance matrix ***Σ*** = [*σ_ij_* ], where *σ_ii_* = 1 for *i*, *j* = 1, …, *M* (that is, ***Σ*** is a correlation matrix). Suppose *S* is a random variable independent of ***Z*** such that *ν S* has a chi-square distribution with *ν* degrees of freedom. Then the joint distribution of (*T*_1_, …, *T_M_*)′ = (*Z*_1_/*S*, …, *Z_M_*/*S*)′ is called a multivariate t-distribution with parameters ***Σ*** and *ν*. A one-sided upper equicoordinate *p* × 100 percentage point *g* is such that

$$\mathrm{Pr}\left\{\underset{1\leq i\leq M}{\max}T_{i}\leq g\right\}=p,$$

and a two-sided upper equicoordinate *p* × 100 percentage point *h* is such that

$$\mathrm{Pr}\left\{\underset{1\leq i\leq M}{\max}\left|T_{i}\right|\leq h\right\}=p.$$

Freeman, Kuzmack, and Maurice [63] tabulated percentage points *g* to, 3D, for *M* = 2, and to, 2D, for *M* = 3, 4, 5, for *p* = 0.95, *ν* = (*M* + 1)*k* for *k* = 9(10)99, 199, 499, and the following correlation structure: *ρ_ij_* = −1/2 for |*i* − *j*| = 1 and *ρ_ij_* = 0 for |*i* − *j*| > 1, where 1 ≤ *i*, *j* ≤ *M*. Freeman and Kuzmack [64] tabulated percentage points *g*, to 2D, for the same correlation structure for *M* = 5(2)9, 9(5)29, *p* = 0.90, 0.95, 0.99 and *ν* = (*M* + 1)*k* for *k* = 9, 19, 49, 99, 499, using Monte Carlo sampling.

Dunn, Kronmal, and Yee [65] computed, using Monte Carlo sampling, probabilities Pr{max_1≤_ *_i_*_≤_ *_M_* |*T_i_*|≤ *h*} = *p*, to 4D, for *M* = 2(2)20, *ρ* = 0.0(0.1)0.9, *h* = 0.2(0.2)6.0, and *ν* = 4(2)12, 12(4)24, 30, ∞.

For the bivariate case *M* = 2, Krishnaiah, Armitage, and Breiter [66] tabulated probabilities Pr{max_1 ≤_ *_i_*_≤_ *_M_ T_i_* ≤ *g*} = *p*, to 6D, for ±*ρ* = 0.0(0.1)0.9, *g* = 1.0(0.1)5.5, and *ν* = 5(1)35. Also for *M* = 2, Krishnaiah, Armitage, and Breiter [67] tabulated probabilities Pr{max_1 ≤_ *_i_*_≤_ *_M_*|*T_i_*| ≤ *h*} = *p*, to 6D, for |*ρ*| = 0.0(0.1)0.9, *h* = 1.0(0.1)5.5, and *ν* = 5(1)35.

Tong [68] tabulated percentage points *g* for the following correlation structure: *ρ_ij_* = 1 for *i* = *j*, *ρ_ij_* = 1/2 for *i* ≠ *j* and 1 ≤ *i*, *j* ≤ *m* or *m* < *i*, *j* ≤ *M*, *ρ_ij_* = −1/2 for 1 ≤ *i* ≤ *m* and *m* < *j* ≤ *M* or 1 ≤ *j* ≤ *m* and *m* < *i* ≤ M where *m* = *M*/2 if *M* is even and *m* = (*M* + 1)/2 if *M* is odd. His Table 1 gives *g*, to 7D, for *M* = 1(1)10, 10(2)20, *p* = 0.50, 0.75, 0.90, 0.95, 0.975, 0.99 and degrees of freedom *ν* = ∞. His Table 2 gives *g*, to 5D, for *M* = 2(1)6, 6(2)12, 12(4)20, *p* = 0.50, 0.75, 0.90, 0.95, 0.975, 0.99 and degrees of freedom *ν* = 5(1)10,10(2)20, 20(4)60, 60(30)120.

Trout and Chow [69] tabulated two-sided nonequicoordinate *p* × 100 percentage points of trivariate (*M* = 3) t-distribution with non-singular correlation matrix

$$\Sigma \left[\begin{array}{ccc} 1.0 & \rho_{12} & \rho_{13}\\ \rho_{12} & 1.0 & \rho_{23}\\ \rho_{13} & \rho_{23} & 1.0 \end{array}\right].$$

They tabulated *d*, to 2D, where

$$\int_{-d}^{d}\int_{-ad}^{ad}\int_{-bd}^{bd}f(t_{1},t_{2},t_{3}|\Sigma ,v)dt_{1}dt_{2}dt_{3}=p,$$

for *p* = 0.95, *ν* = 5(1)9(2)29, *a* = 0.5(0.1)1.5, *b* = 0.5(0.1)1.5, and a set of 22 triplets (*ρ*_12_, *ρ*_13_, *ρ*_23_), where *ρ_ij_* = 0.0, 0.1, 0.5, 0.9, (*i* ≠ *j*, 1 ≤ *i*, *j* ≤ 3).

Dutt [70] tabulated the probabilities Pr{max_1≤_ *_i_*_≤_ *_M_ T_i_* ≤ *g*} = *p*, to 6D, for *g* = 0.0(0.5)2.0, 2.0(1.0)4.0, and *ν* = 8(4)40, and ∞: for *M* = 3 with (*ρ*_12_, *ρ*_13_, *ρ*_23_) = (0.3, 0.5, 0.7), and (0.1, 0.3, 0.5); and for *M* = 4 with (*ρ*_12_, *ρ*13, *ρ*_14_,*ρ*_23_, *ρ*_24_, *ρ*_34_) = (0.05, 0.10, 0.15, 0.25, 0.60, 0.80), and (0.25, 0.35, 0.50, 0.60, 0.65, 0.70).

Bechhofer and Dunnett [71] tabulated one-sided and two-sided upper equicoordinate percentage points for *M* = 2(1)16, 16(2)20, degrees of freedom *ν* = 2(1)30, 30(5)50, 60(20)120, 200, ∞, and *ρ* = 0.0(0.1)0.9, and $1/(1+\sqrt{M})$. They tabulate, to 5D, *g* and *h* for *p* = 0.80, 0.90, 0.95, and 0.99. They also tabulate equicoordinate and non-equicoordinate onesided percentage points for block correlation structure. Bechhofer and Dunnett [71] summarize previous tables of percentage points for equicorrelated multivariate normal and t-distributions.

### 4.3 Distribution of the Wilks’s Likelihood Ratio Test Statistic

Schatzoff [72], Pillai and Gupta [73], Lee [74], and Davis [75] tabulate multiplying factors *C* to obtain upper percentage points of the distribution of the Wilks’s Likelihood Ratio Test Statistic – [*n − p −* (1/2) (*m − r* + 1)] logW from the percentage points of the chi-square distribution for multivariate analysis of variance. Muirhead [76] has consolidated these into one large table. Here, *n* is the number of multivariate measurements, *p* is the number of regression parameter vectors, *n* − *p* is the error degrees of freedom, *m* is the dimension of multivariate measurements, and *r* is the degrees of freedom of the general linear hypothesis. Factors for the upper *α* × 100 percent points are tabulated for *α* = 0.100, 0.050, 0.025, and 0.005. The chi-square distribution has *mr* degrees of freedom. The degrees of freedom *n* − *p* − *m* + 1 equal 1(1)10, 10(2)20, 24, 30, 40, 60, 120, and ∞. Pairs (*m*, *r*) are such that *m* = 3(1)10, 12, and *r* ≥ *m*, where *r* is up to 22 for *m* = 3, and 4, *r* is up to 20 for *m* = 5, 6, and 7, and *r* is up to 18, 16, and 14 for *m* = 8, 9, and 10, respectively. Pairs (*m*, *r*) = (6, 11), (6, 13), and (10, 13) are excluded. For *r* ≤ *m* make the substitutions *m* → *r*, *r* → *m*, and *n* − *p* → *n* + *r* − *p* − *m*.

### 4.4 Dirichlet Distribution—Type 1

Sobel, Uppuluri, and Frankowski [77] tabulated, to 10D, the incomplete Dirichlet integral of Type 1:

$$\begin{array}{l} I_{p}^{\left(b\right)}(r,\;n)=\;\\ \begin{array}{c} \frac{\Gamma (n+1)}{\Gamma^{b}(r)\Gamma (n-br+1)}\\ \times \int_{0}^{p}\ldots \int_{0}^{p}{\left(1-\sum_{i=1}^{b}x_{i}\right)}^{n-br}\overset{b}{\underset{i=1}{\Pi }}x_{i}^{r-1}dx_{i} \end{array} \end{array}$$

for *p* = 1/*b*, *b* = 2(1)10, *r* = 1(1)10, and *n* ≥ *br*. This represents $\text{Pr}\left\{\cap_{i=1}^{b}\left(X_{i}\leq p\right)\right\}$ where *X*_1_,…, *X_b_* have a joint Dirichlet distribution with the specified parameters. Also tabulated, to 10D, are values of $I_{p}^{\left(b\right)}(r,\;n)$ for *p* =1/*j*, *j* = *b*+1(1)10, *b* =1(1)10, and and *r* = 1(1)10. Values of *n* are given to, 2D, for which $I_{p}^{\left(b\right)}(r,\;n)$ for *M* = 0.75, 0.90, 0.95, 0.975, 0.99, 0.999, 0.9999, *p* = 1/*j*, *j* = *b* + 1(1)20, *b* = 1(1)10 and *r* = 1(1)10. Additional tables are given for Generalized Stirling Numbers and for the sample size required for occupancy problems in multinomial distributions.

### 4.4 Dirichlet Distribution—Type 2

Sobel, Uppuluri, and Frankowski [78] tabulated the incomplete Dirichlet integrals of Type 2:

$$\begin{array}{l} C_{a}^{\left(b\right)}(r,\;m)=\frac{\Gamma (m+br)}{\Gamma^{b}(r)\Gamma (m)}\\ \;\times \int_{0}^{a}\ldots \int_{0}^{a}\frac{\Pi_{i=1}^{b}x_{i}^{r-1}dx_{i}}{{\left(1+\sum_{i=1}^{b}x_{i}\right)}^{m+br}}, \end{array}$$

$$\begin{array}{l} D_{a}^{\left(b\right)}(r,\;m)=\frac{\Gamma (m+br)}{\Gamma^{b}(r)\Gamma (m)}\\ \;\times \int_{a}^{\infty }\ldots \int_{a}^{\infty }\frac{\Pi_{i=1}^{b}x_{i}^{r-1}dx_{i}}{{\left(1+\sum_{i=1}^{b}x_{i}\right)}^{m+br}}. \end{array}$$

The lower tail integral $C_{a}^{\left(b\right)}(r,\;m)$ is tabulated, to 8D, for the parameters: {*r* = 1(1)10, *b* = 1(1)15, *m* = 1(1)15, *a* = 1(1)5, and *a*^−1^ = 2(1)5} and {*r* = *m*, *b* = 1(1)10, *a* = 0.40(0.10)0.60, *a* = 0.60(0.05)0.80, and *a*^−1^ = 3(1)10}.

The upper tail integral $D_{a}^{\left(b\right)}(r,\;m)$ is tabulated, to 8D or 10D, for the parameters: {*r* = 1(1)10, *b* = 1(1)15, *m* = 1(1)15, *a* = 1(1)5, and *a*^−1^ = 2(1)5}, {*r* = *m*, *b* = 1(1)10, *a* = 3(1)10, *a*^−1^ = 0.40(0.10)0.60, and *a*^−1^ = 0.60(0.05)0.80}, {*m* = *r* + 1, *b* = 1(1)10, *a* = 3(1)10, *a*^−1^ = 0.40(0.10)0.60, and *a*^−1^ = 0.60(0.05)0.80}, {*r* = *m*, *b* = 1(1)10, *a* = 0.40(0.10)0.60, *a* = 0.60(0.05)0.80, and *a*^−1^ = 3(1)10}, {*m* = *r* + 1, *b* = 1(1)10, *a* = 40(0.10)0.60, 0.60(0.05)0.80, and *a*^−1^ = 3(1)10}, {*m* = *r* + 1, *r* = 1(1)200, *a* = 1, and *b* = 1(1)10}, and {*m* = *r* + 2, *r* = 1(1)200, *a* = 1, and *b* = 1(1)10}. Values of *a* for which $D_{a}^{\left(b\right)}(r,\;m)$ = *δ* are tabulated for *δ* = 0.75, 0.95, 0.975, 0.99, 0.995, 0.999, *r* = 1(1)50, and *b* = 1(1)10. A table for expected waiting time in multinomial problems is also given.

### 4.6 Zonal Polynomials

Probability density functions and moments of many multivariate distributions can be evaluated using zonal polynomials. Parkhurst and James [79] tabulate zonal polynomials of order 1 through 12 in terms of sums of powers and in terms of elementary symmetric functions.

### 4.7 Distributions of the Largest and Smallest Eigenvalues of Matrices of Sample Quantities

Heck [80] charts some upper percentage points of the distribution of the largest eigenvalue of certain matrices of sample quantities from multivariate normal distribution. Edelman [81] tabulates expected values of the smallest eigenvalue of random matrices of Wishart type.

## 5. Summary

This article is a survey of the tables of probability distributions published about or after the publication in 1964 of the Handbook of Mathematical Functions, edited by Abramowitz and Stegun. The abstracts presented here have been verified from the original sources. Many of the distributions referenced here are implemented in commercial or publicly-available software systems.
